# The flight of the hornbill: drift and diffusion in arboreal avian movement

**DOI:** 10.1038/s41598-021-84074-3

**Published:** 2021-03-10

**Authors:** Ankit Vikrant, Janaki Balakrishnan, Rohit Naniwadekar, Aparajita Datta

**Affiliations:** 1 School of Natural Sciences and Engineering, National Institute of Advanced Studies (N.I.A.S.), Indian Institute of Science Campus, Bangalore, 560012 India; 2 Nature Conservation Foundation, 1311, Amritha, Vijayanagar 1st Stage, Mysore, 570017 India; 3 Present Address: Department of Space, Earth and Environment, Chalmers University of Technology, Maskingränd 2, 412 58 Gothenburg, Sweden

**Keywords:** Statistical physics, Ecological modelling, Behavioural ecology, Animal migration, Ecology, Physics

## Abstract

Capturing movement of animals in mathematical models has long been a keenly pursued direction of research^1^. Any good model of animal movement is built upon information about the animal’s environment and the available resources including whether prey is in abundance or scarce, densely distributed or sparse^2^. Such an approach could enable the identification of certain quantities or measures from the model that are species-specific characteristics. We propose here a mechanistic model to describe the movement of two species of Asian hornbills in a resource-abundant heterogenous landscape which includes degraded forests and human settlements. Hornbill telemetry data was used to this end. The birds show a bias both towards features of attraction such as nesting and roosting sites as well as possible bias away from points of repulsion such as human presence. These biases are accounted for with suitable potentials. The spatial patterns of movement are analyzed using the Fokker–Planck equation, which helps explain the variation in movement of different individuals. Search times to target locations were calculated using first passage time equations dual to the Fokker–Planck equations. We also find that the diffusion coefficients are larger for breeding birds than for non-breeding ones—a manifestation of repeated switching of directions to move back to the nest from foraging sites. The degree of directedness towards nests and roosts is captured by the drift coefficients. Non-breeding hornbills show similar values of the ratio of the two coefficients irrespective of the fact that their movement data is available from different seasons. Therefore, the ratio of drift to diffusion coefficients is indicative of an individual’s breeding status, as seen from available data. It could possibly also characterize different species. For all individuals, first passage times increase with proximity to human settlements, in agreement with the premise that anthropogenic activities close to nesting/roosting sites are not desirable.

Animal movement is a complex process where complexity of motion increases as the environment around the animal becomes more heterogeneous. Most mathematical analyses used to understand animal movement focus on either roughly predicting the movement path (Lagrangian Approach) or quantifying how the probability of finding an animal at some locations changes over time (Eulerian Approach)^3^. The Langevin equation is arguably the most basic equation that can be used to describe the movement path of an animal where every step has deviations driven by some type of random noise. Most models that employ the Langevin equation to simulate animal movement use expressions of polynomial type in the drift term^4,5^. Though these seem to be useful in many cases, they fail to capture the observed dynamics when the landscape is highly complex and heterogeneous. A particular case where these would be insufficient is when there are multiple features in the landscape that influence an animal’s movement. A polynomial drift term would mean that as the animal moves away from the location of bias, the attraction would also increase since these terms represent forces and are proportional to some positive power of distance. For cases of bias towards multiple features, this framework implies that at any given time, an animal would be more strongly attracted to locations that are farther from it. Other functions of distance have been used more effectively for the drift term, but their use within the animal movement literature is currently very limited^6^.

Another important aspect about animal movement is search time to any site in an animal’s home range. It is hard to disentangle information about this from empirical data, and therefore not many studies have addressed it convincingly. Mathematical models that attempt to do so face computational challenges depending upon the complexity of the landscape being studied.

Based on various theoretical considerations and inputs from empirical data for large forest hornbill species from North-East India, we propose a robust framework to model movement in heterogeneous landscapes. In doing so, we try to address the issues mentioned above and uncover some general features that might be useful for various studies elsewhere. Hornbills display a diverse range of movements ranging from a high bias towards a single nest site during the breeding season to utilizing multiple roost sites in their non-breeding phase. This provides a good study system for our model that captures important aspects of movement without relying on fine-scale resource abundance data.

We analysed the telemetry data recorded by two of us (RN and AD) of six tagged hornbills (five Great hornbills *Buceros bicornis* and one Wreathed hornbill *Rhyticeros undulatus*) in the Pakke Tiger Reserve in the northeastern state of Arunachal Pradesh in India. Our telemetry did not encompass the entire breeding cycle which for the hornbills lasts till July-August. The length of the nesting cycle is from March to July—around 120 days for Great Hornbills and 130 days for the Wreathed Hornbill. Details on the nature and collection of the data and study area are given in Methods.

## Proposed model

### A mathematical model to simulate movement

For ‘attracting features’, such as nesting or roosting sites, we employ potential terms that are logarithmic in distance. Logarithmic potentials have been employed in diffusion models^7^ such as those involving long-range interactions^8^. The forces due to these are inversely proportional to distance from the features. Given a choice between locations, an animal would invariably drift towards ones that are closer. Additionally, they also command some influence for longer distances. We did consider alternatives such as a potential that corresponds to an inverse squared force but it diminishes much faster as the distance to the source increases. The ‘repulsive features’ such as human dominated areas are incorporated using Gaussian type potentials that would have an influence only when the animal is close to them. Such forces fall off exponentially fast as one goes away from the source location.

The corresponding Langevin equations can be written as:

1 $$\begin{aligned} \frac{dx}{dt}= & {} -\gamma \sum _{i}\frac{ 2\alpha \times (x - x_{i})}{(x - x_{i})^2 + (y - y_{i})^2} \nonumber \\&+\,\gamma \sum _{j}\Big ((x-x_{j}) e^{-((x-x_{j})^2 + (y-y_{j})^2)}\Big ) + \root 2 \of {2D}\xi _x(t) \end{aligned}$$

2 $$\begin{aligned} \frac{dy}{dt}= & {} -\gamma \sum _{i}\frac{ 2\alpha \times (y - y_{i})}{(x - x_{i})^2 + (y - y_{i})^2} \nonumber \\&+\,\gamma \sum _{j}\Big ((y-y_{j}) e^{-((x-x_{j})^2 + (y-y_{j})^2)}\Big ) + \root 2 \of {2D}\xi _y(t) \end{aligned}$$

where *x* and *y* denote the coordinates of an animal’s location. ($x_{i}$, $y_{i}$) and ($x_{j}$, $y_{j}$) denote locations of *i* attracting and *j* repelling features respectively. We only choose nests as points of attraction for breeding hornbills since their diurnal movements are strongly centred around the nests. The white noise terms $\xi _x$ and $\xi _y$ are Gaussian in nature and delta correlated—which means that no correlations exist between the noise values at different instances of time. $\gamma$ and *D* denote the drift and diffusion coefficients respectively. The drift coefficient $\gamma$ represents the directedness of motion, which could be interpreted as strength of bias towards/against certain features in the landscape. In contrast, *D* quantifies the strength of random undirected motion. The force term with coefficient $-\gamma$ results from negative gradient of the logarithmic potential, whose choice we explained earlier:

3 $$\begin{aligned} U = \gamma \sum _{i} \log \left\{ (x - x_{i})^2 + (y - y_{i})^2 \right\} ^{\alpha } \,\,\,. \end{aligned}$$

The value of $\alpha$ is determined from calculation of first passage times of the birds (discussed in the following section) and comparison of the values so obtained with observational (telemetry) data. We find that $\alpha$ = 8 gives biologically sensible first passage times for hornbills (see “Calculating First Passage Times” in Methods section, Table 3 and Supplementary Tables 1, 2). If one observes an animal’s movement for a very long time, the probability of finding the animal would decrease more drastically away from a central feature for lower values of $\alpha$. Such variations are captured by the steady-state probability distributions of space-use that we describe in the following section.

### Fokker–Planck methods

Although the Langevin equations can generate trajectories of movement, the corresponding simulations need to be run for very long times to infer reliable information about spatial use. The time steps are further much smaller than the frequency of data recorded by the GPS. The step-lengths thus generated from simulated trajectories do not lend themselves to comparison against those from the recorded data. A convenient alternative is to solve a Fokker–Planck equation which has a direct correspondence with the Langevin equations. For our model, this takes the form:

4 $$\begin{aligned} \frac{ \partial P(x,y,t)}{\partial t}&= \frac{\partial }{\partial x} \left\{ F_x + D \frac{\partial }{\partial x} \right\} P(x,y,t) \nonumber \\&\quad +\, \frac{\partial }{\partial y} \left\{ F_y + D \frac{\partial }{\partial y}.\right\} P(x,y,t) \end{aligned}$$

where

5 $$\begin{aligned} F_x&= -\gamma \sum _{i} \frac{ 2 \alpha \times (x - x_{i})}{(x - x_{i})^2 + (y - y_{i})^2} \nonumber \\&\quad+\, \gamma \sum _{j} (x - x_{j}) \times e^{-( (x - x_{j})^2 + (y - y_{j})^2)} \nonumber \\ F_y&= -\gamma \sum _{i} \frac{ 2 \alpha \times (y - y_{i})}{(x - x_{i})^2 + (y - y_{i})^2} \nonumber \\&\quad+ \gamma \sum _{j} (y - y_{j}) \times e^{-( (x - x_{j})^2 + (y - y_{j})^2)} \end{aligned}$$

The Fokker–Planck equation describes the evolution of the probabilities of occurrence over a given region. The probability distribution eventually reaches a ‘steady state’ which captures the long-term occurrence probabilities for a given bird, and it does not change beyond this point in time. This steady-state probability distribution can be computed by setting the time derivative term to zero in Eq. (4). The numerical solution of the Fokker–Planck equation involves discretizing the spatial derivatives involved. The steady state probability distribution is consequently obtained on a spatial domain of discretized grids.

Interestingly, Giuggioli *et al.*^9^ considered logarithmic potentials in their work on home range estimation, where an exponent of 8 was found to have a very similar steady state distribution to that from a harmonic potential. Harmonic potential has been utilized in analyzing home ranges of *Peromyscus maniculatus*^10^.

Using the steady-state solution of the Fokker–Planck equation, we compute the mean square displacement averaged over different possible starting locations using the steady state distribution. A discrete version of the mean-square displacement (*MSD*) can be defined as:

6 $$\begin{aligned} MSD = \sum _i^N \langle (x - x_i)^2 + (y - y_i)^2 \rangle P_{0}(x_i,y_i) \end{aligned}$$

where $P_0(x_i,y_i)$ is the distribution of starting locations $x_i$ and $y_i$ from where displacements are calculated. The inner angular brackets represent a similar weighted average of the mid-points of all grids over the steady-state probability distribution $P_{\text {st}}(x,y)$. Many of the grids that we define to perform simulations lie outside the known home range of the birds. The probability of choosing a starting location is defined using a Gaussian distribution centred around the nest or the most visited roost site.

The square root of the *MSD* defines a characteristic length scale. This could be interpreted as home range length when the steady state distribution is computed over an infinite extent^9^. A logarithmic potential does not lend itself to such computations since it decays much more slowly such that the characteristic length continues to grow with the size of the area considered. We evaluate the characteristic length scale (*L*) on a domain that is not much larger in size compared to the observed home range.

We also calculate *L* from empirical data by using the probability of occurrence over space inferred from two-dimensional histograms of location data. The *MSD* in this case is evaluated in the same vein as above but now the displacements from initial locations are weighted over the probabilities of occurrence derived from the histograms. Since these probabilities are only available for each grid, we choose only the mid-points of grids as possible locations to find the result. The starting locations are chosen from a uniform distribution over the mid-points of the grids. This is definitely a crude way of evaluating *L* but it does give us some way of comparing our numerical solutions against data. Finding a joint-probability distribution over the two dimensions would have been ideal but it is complicated by the fact that the distribution over space is multi-modal owing to multiple roosts for some hornbills. When inferring MSD from the location coordinates directly, it increases before saturating as the sampling frequency is decreased. For very high sampling frequency (or very small time intervals), diffusion effects dominate which leads to an almost linear increase in MSD. The effects of drift are more prominent compared to diffusion for lower sampling frequencies which marks the saturation of the MSD values^10^.

### A first-passage time model for heterogeneous environments

The temporal information about an animal’s whereabouts is highly scrambled in the data. An important quantity of interest that could be extracted from movement data is the search time to reach a given target. A very useful measure of search times is the ‘first passage time’. Very generally, first passage time is the time taken for a given state variable to reach a particular value. In the case of animal movement, it can be interpreted as the time taken to reach a particular target location. McKenzie et al.^11^ derived an interesting first passage time model which had a direct correspondence with a Fokker–Planck equation. We use the prescription of Moorcroft et al.^12,13^ to estimate the drift and diffusion coefficients. This assumes a movement kernel that is a product of exponential distribution of step lengths and von Mises distribution for the turning angles. (This may be seen in the “goodness of fit tests” section in Methods where we assess fit of our data to claimed distributions.) It can be expressed as:

7 $$\begin{aligned} K({\mathbf{X}} ,{\mathbf{X}}' ,\tau )=\, & {} \frac{1}{\rho } f_\tau (\rho ) k_\tau (\phi ) \end{aligned}$$

8 $$\begin{aligned} {\rm{where}}\,\,\,\,\,\,\,\,\,\,\,\, \,f_\tau (\rho )=\, & {} \lambda e^{-\lambda \rho }\end{aligned}$$

9 $$\begin{aligned} k_\tau (\phi )=\, & {} \frac{1}{2 \pi I_0(\kappa _\tau )} \exp [\kappa _\tau \cos (\phi )] \end{aligned}$$

Here, ${\mathbf{X}}$, ${\mathbf{X}}'$ denote the current and previous locations respectively, *f* is the exponential distribution of step lengths $\rho$ with rate parameter $\lambda$ and mean $\bar{\rho }_{\tau } = 1/\lambda$, and $k_{\tau }$ is the von Mises distribution of turning angles $\phi$. $\tau$ refers to the time taken to complete a given step. The turning angles are computed with respect to the nest/roost sites. $\kappa _\tau$ is the concentration parameter of the von Mises distribution which signifies the departure from a uniform distribution of movement directions. The normalizing factor $I_0(\kappa _\tau )$ is a modified Bessel function of the first kind and of zeroth order. The drift and diffusion coefficients can be reliably estimated as:

10 $$\begin{aligned} \gamma= & {} \lim _{\tau \rightarrow 0} \frac{\bar{\rho }_{\tau } \kappa _\tau }{2\tau } \end{aligned}$$

11 $$\begin{aligned} D= & {} \lim _{\tau \rightarrow 0} \frac{\bar{{\rho _{\tau }}^2}}{4\tau } \end{aligned}$$

Employing the formalism in McKenzie^11^ to derive the equation for the first passage time *T*, we obtain the following equation:

12 $$\begin{aligned}&\gamma \sum _{i} \left\{ \frac{ 2\alpha \times ({\mathbf{X}} - {\mathbf{X}} _{i})}{(x - x_{i})^2 + (y - y_{i})^2} \right\} \cdot \nabla T \nonumber \\&\quad -\, \gamma \sum _{j} \left\{ ({\mathbf{X}} - {\mathbf{X}} _{j}) e^{-( (x - x_{j})^2 + (y - y_{j})^2)} \right\} \cdot \nabla T \nonumber \\&\quad +\, D \nabla ^2 T + 1 = 0 \end{aligned}$$

The terms in dot product with $\nabla T$ are simply the drift coefficients with spatial dependence.

McKenzie et al.^11^ had a simpler version of the first passage time equation that only accounted for bias towards the home range centre. The authors mention that the task of solving the first passage time equation is computationally harder with terms that account for more complex types of heterogeneities. We transform the partial differential equation in (12) into polar coordinates which simplifies the process of solving it (see First Passage Time calculation in Methods). The first passage times obtained from this solution also help us fix the value of $\alpha$ in the equation above and subsequently in the logarithmic potential in (3), and in Eqs. (1) and (2). On performing this analysis for different hornbills, we see that $\alpha$ = 8 works very well for them irrespective of the species and distribution of heterogeneities around them (see First Passage Time calculation in Methods). First passage times are calculated from the roosting/nesting site that lies closest to the home range centre. In case of GHNBr2, we calculate the first passage times from the approximate home range centre where no roosts exist. This ensures that most points considered for computations lie within the actual extent of the bird’s recorded locations. We used the Minimum Convex Polygon method to estimate the approximate home range centre^14^. This helped in identifying a location for each bird—which was a roost/nest in most cases—from where first passage times were subsequently computed. The method used for home range estimation is not relevant in the context of our proposed model and results presented, and therefore we do not consider other alternatives.

## Results

**Figure 1:**
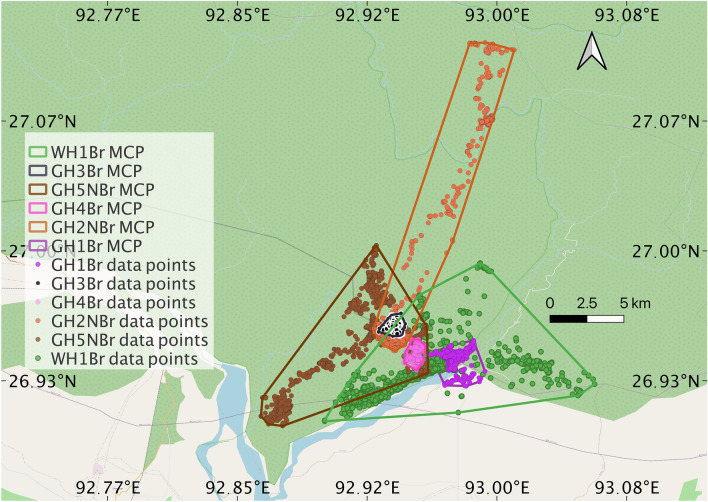
Data points and corresponding 100% Minimum Convex Polygons for different hornbills with respect to the landscape.

**Figure 2:**
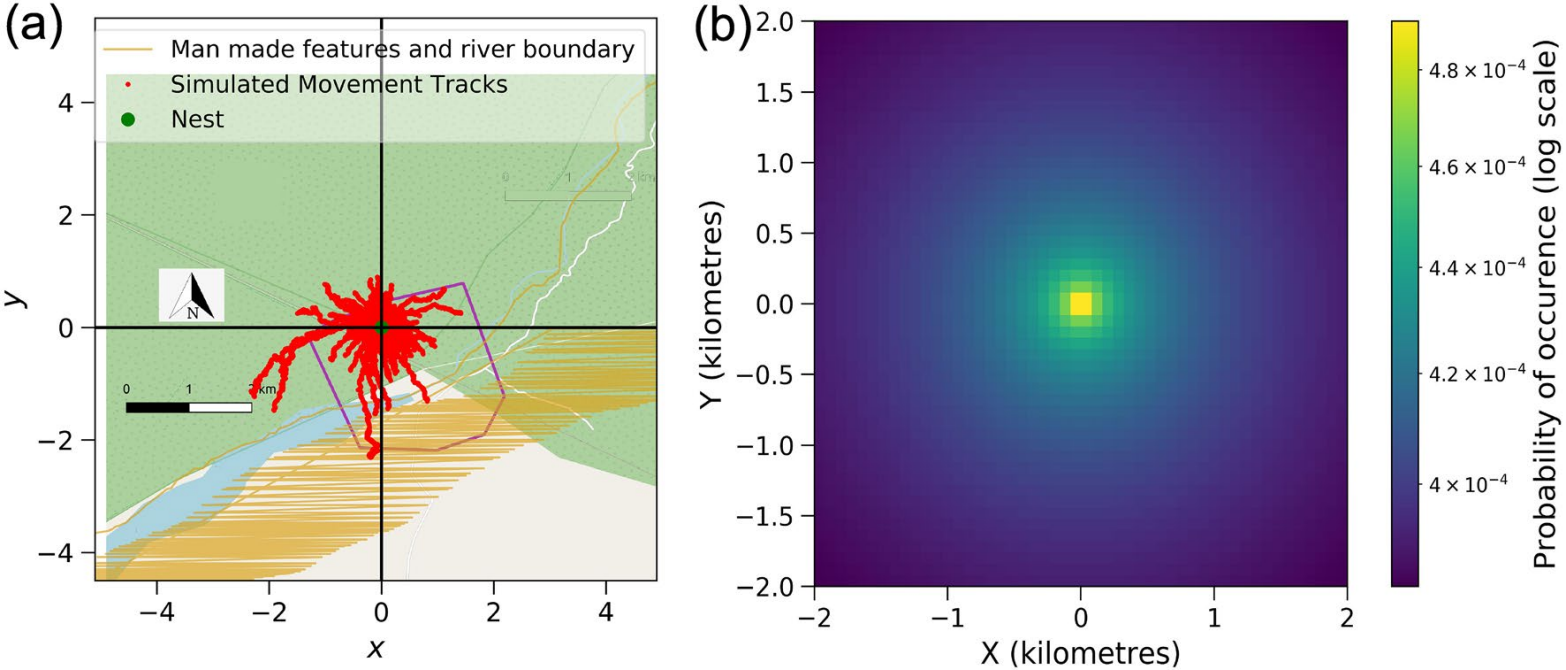
(**a**) Simulated movement points for breeding Great hornbill GH1Br generated with 7500 time steps from the Langevin equation. (**b**) Corresponding probability map of spatial use from Fokker–Planck equation. Also see Supplementary video SI Fig 1.

Movement data was gathered using e-obs tags from 20 to 81 days of six hornbill individuals named GH1Br, GH3Br, GH4Br, GH2NBr, GH5NBr and WH1Br by the observers. This is represented in Fig. 1 and in Table 1. Figure 2a shows movement simulation for the Great hornbill GH1Br using the Langevin equation and its corresponding probability map (Fig. 2b) of spatial use from the Fokker–Planck equation. Results for the other hornbills are presented in the Section “Probability distribution of space use of territories of other hornbills'' in Methods. In Table 2, we compare the characteristic length *L* calculated from data and the Fokker–Planck equation.

**Table 1:** Estimated drift ($\gamma$) and diffusion (*D*) coefficients for hornbills.

Hornbill code	Species	Tagging Period	Season	Status	$\gamma$	D	$\gamma /D$
GH1Br	GH	2 Mar–19 May, 2015 (79 days)	B	B male	0.455	0.060	7.58
GH3Br	GH	17 Feb–08 Apr, 2016 (52 days)	B	B male	0.289	0.042	6.88
GH4Br	GH	25 Feb-15 Mar, 2016 (20 days)	B	B male	0.348	0.085	4.09
GH2NBr	GH	23 Nov’15 -15 Jan’16 (54 days)	NB	NB male	0.376	0.041	9.17
GH5NBr	GH	5 Mar–10 May, 2016 (67 days)	B	NB male	0.317	0.034	9.32
WH1Br	WH	29 Mar–17 Jun, 2015 (81 days)	B	B male	1.865	0.575	3.24

B denotes breeding, NB denotes non-breeding, GH stands for Great hornbill, WH for Wreathed hornbill. GH2NBr (an adult non-breeding male) was tagged in the non-breeding season, the younger non-breeding male GH5NBr was tagged in the breeding season.

**Figure 3:**
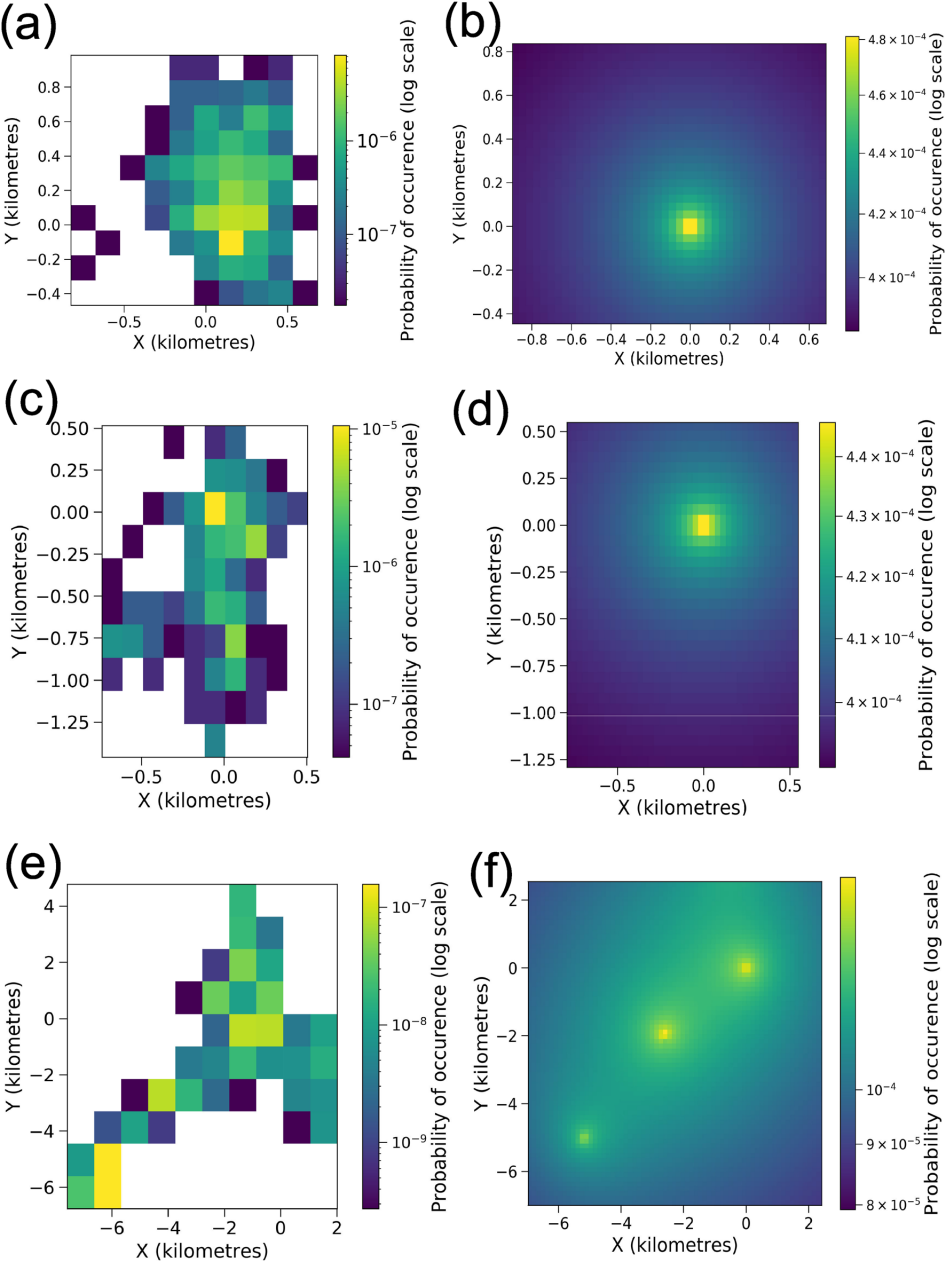
The probability of space use over the territories from empirical data against the steady-state probability distributions from Fokker–Planck equation. The details are as in Fig. 11 for (**a**, **b**) GH3Br, (**c**, **d**) GH4Br, (**e**, **f**) GH5NBr.

**Figure 4:**
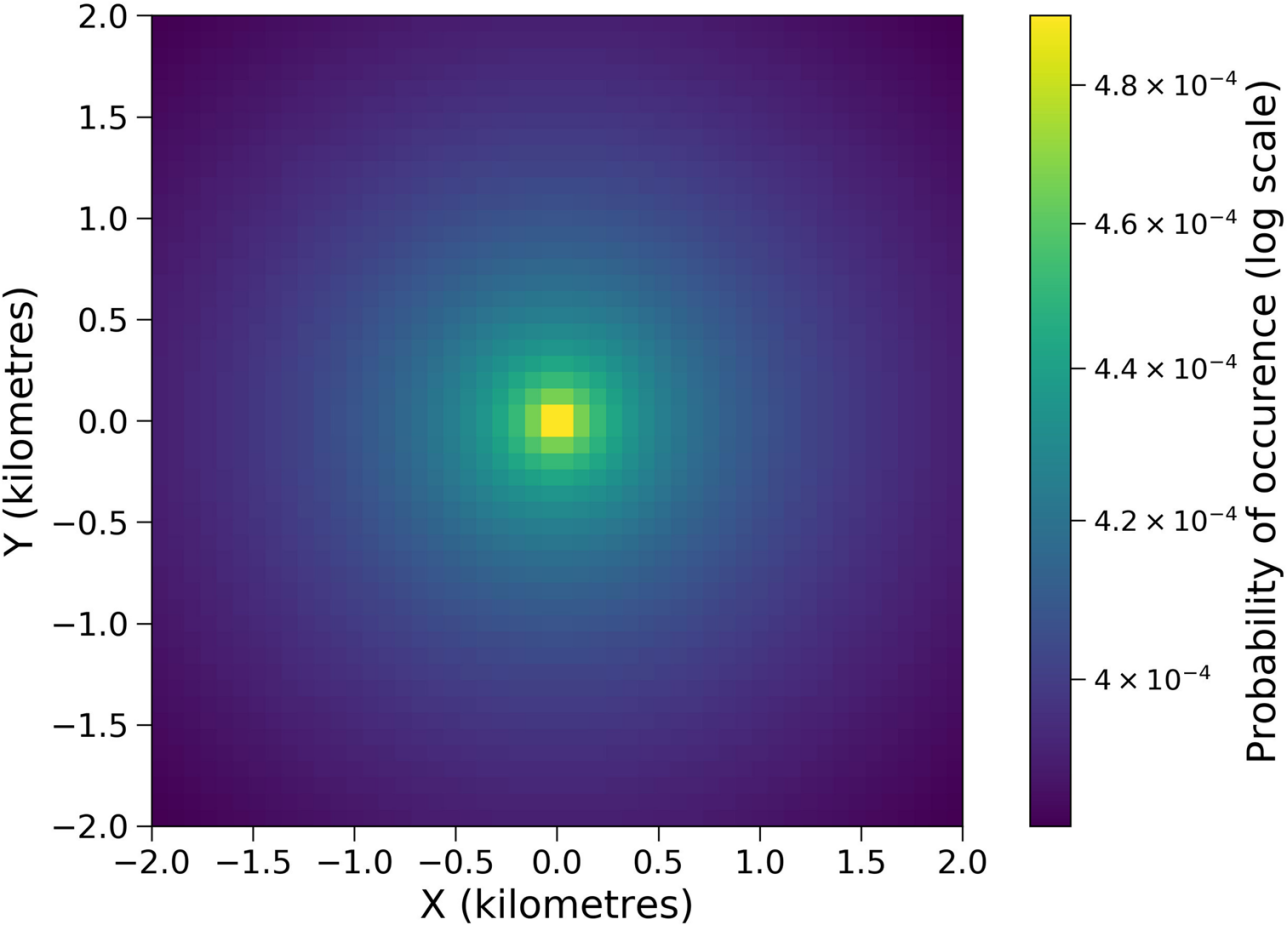
The steady-state probability distribution from the Fokker–Planck equation for GH1Br without using the repulsive terms. The probabilities are very similar when compared to Fig. 11b that includes data for repulsive locations.

The steady-state probability distributions in Figs. 2 and 3, and Fig. 11 in the Methods section were generated numerically using the package ‘fplanck’ in python. The repulsive terms have a very weak influence on the probability distribution of space use. These distributions could very well be approximated through analytical calculations for breeding hornbills, whose activity is strongly centred around a single nest (Fig. 4). The influence of the repulsive terms affects the steady-state probability distributions only at fine spatial scales since the space use is much more strongly influenced by nests over longer times. However, search times are significantly affected by these repulsive features (See Results Section “Temporal patterns in hornbill movement”).

The steady-state probabilities fell off very slowly away from the points of attraction. This feature is ensured by the logarithmic potential that we choose. This is especially helpful to study movement patterns of birds such as WH1Br, which makes some rare trips to regions far away from its nest. The probability of occupying these far off areas is extremely low as the empirical data shows (Fig. 11e). The results from Fokker–Planck equation also yield very small but non-zero probabilities at such distances (Fig. 11f). Other potentials like the harmonic potential are useful to capture movements that can be represented by simple random walks^10^, but they are sub-optimal when the probability distributions of space use have long tails.

**Figure 5:**
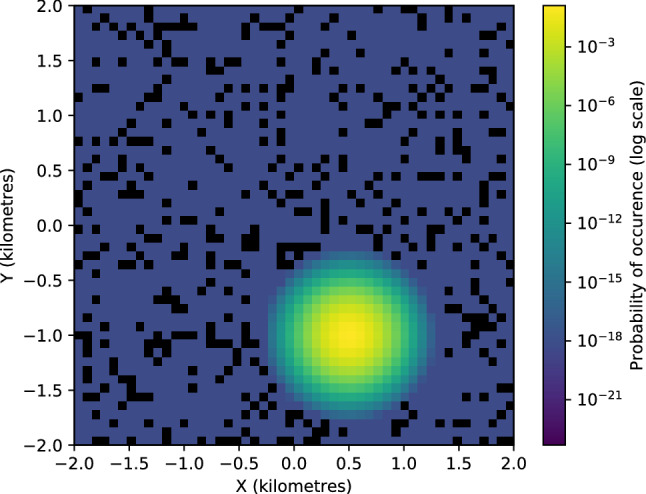
Steady-state probability distribution computed for GH1Br using harmonic potential that scales as $x^2$. For this case, we placed an additional hypothetical roost site at ($X=1$ km, $Y=2$ km) apart from the nest at the origin. The probability distribution is centred around the centroid of the two roosts. Also, note that the probability is more evenly spread in the region where it is not close to 0 (represented by the yellow region).

As we previously posited, logarithmic potentials can also explain movement around multiple roosts, which is the case for GH2NBr and GH5NBr. Our solutions show that the steady-state probabilities are appropriately distributed around the roosts (Figs. 3f and 11d). Potentials that scale with some positive power of distance would fail here. The spatial probability would peak around the centroid of roosts rather than at the roosts themselves (Fig. 5) which does not match with empirical evidence.

**Table 2:** An approximation of characteristic length scales from data and numerical solutions.

Hornbill	L from data (m)	L from steady-state distribution (m)	Percent error
GH1Br	1386.68	1630.43	17.56
GH3Br	720.36	755.72	4.91
GH4Br	771.11	970.60	25.87
GH2NBr	9477.95	9730.95	2.67
GH5NBr	6029.41	5580.81	7.44
WH1Br	6424.16	6851.59	6.65

The calculations from empirical data were executed over larger grid sizes that were obtained from the two-dimensional histograms of location data.

The movement model we described was supplemented by knowledge of drift and diffusion coefficients. These coefficients were estimated from the empirical data and their values also unveil some interesting patterns. Table 1 suggests differences in the values of both coefficients between breeding and non-breeding hornbills.

### Breeding status and classification of movement patterns

The breeding birds had higher values of *D*, that quantifies the strength of random undirected motion. We note that the Wreathed hornbill WH1Br which also ranges over a larger area had much higher values of both $\gamma$ and *D* than the other Great hornbills.

The two non-breeding hornbills had very similar values of the ratio of the coefficients $R\equiv \gamma /D$ in spite of the data being from different seasons. The values of *R* were 9.17 and 9.32 for GH2NBr and GH5NBr respectively. Interestingly, we find that while the ratio *R* for the breeding Great hornbills ranged from 4.09 to 7.58, it was about 9.17–9.32 for non-breeding Great hornbills, and was around 3.24 for the breeding Wreathed hornbill. If $R=0$, then the motion is entirely random and there is no bias towards or against any feature. Higher values of *R* indicate that the movement of any individual is more directed than random. Given a certain species, anchoring of the birds to their nests is what makes the ratios different.

### Temporal patterns in hornbill movement

**Figure 6:**
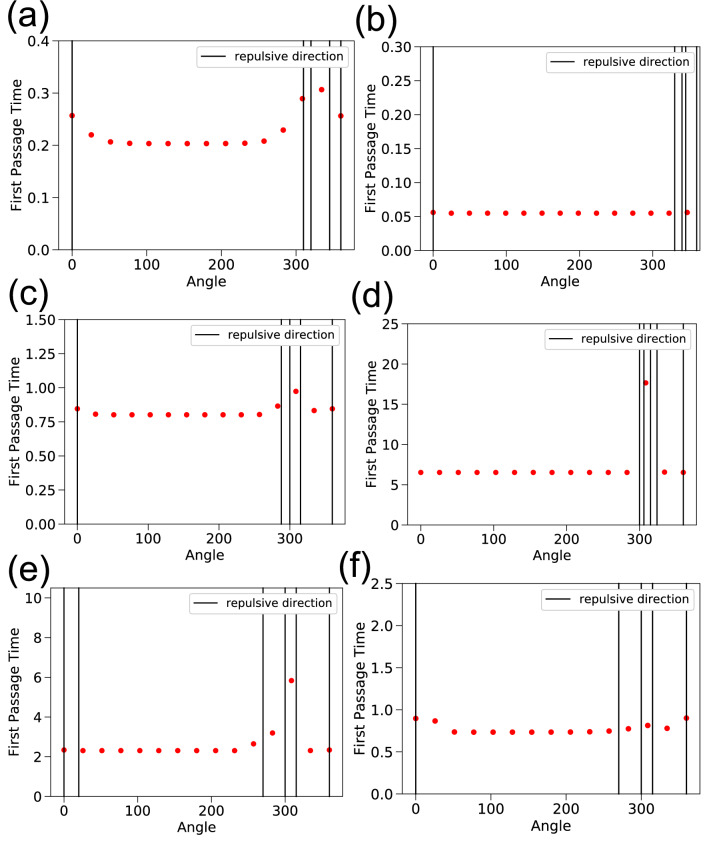
First Passage Times (in hours) for individual hornbills closer to the edge of their home ranges, for different angles. (Vertical) Black lines indicate repulsive directions. Distances *r* from the home range centre are respectively: (**a**) GH1Br, r = 1.8 (**b**) GH3Br, r = 0.74 (**c**) GH4Br, r = 3.16 (**d**) GH2NBr, r = 9.28 (**e**) GH5NBr, r = 5.11 (**f**) WH1Br, r = 7.00 km. Also see Supplementary video SI Fig. 4.

**Figure 7:**
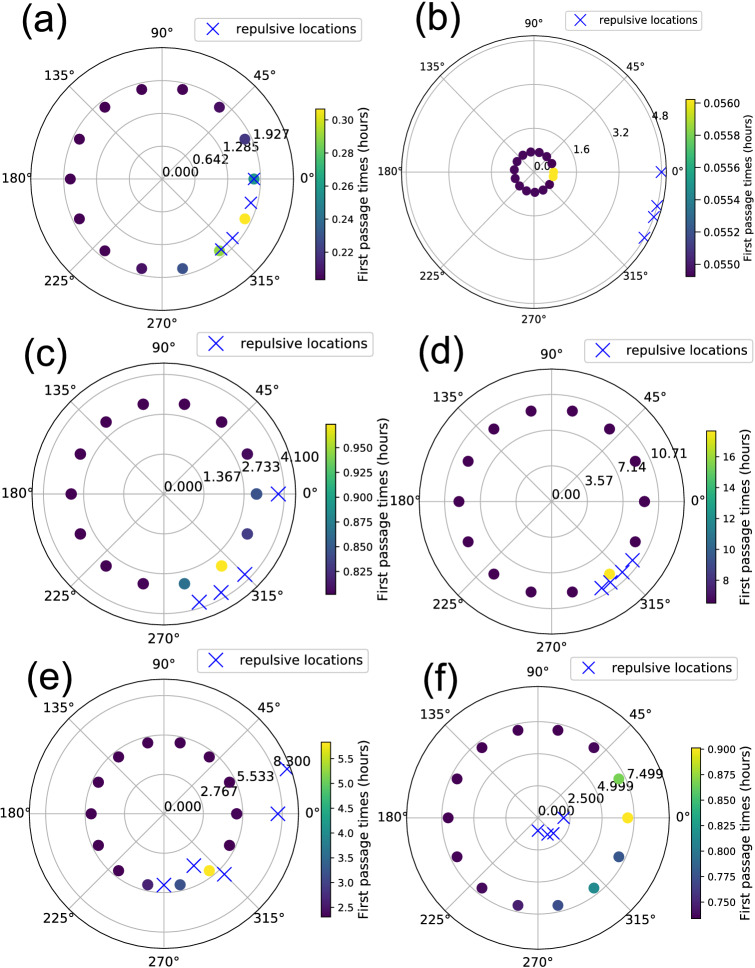
Polar plots depicting the locations considered for calculation of the first passage times. There is a one to one correspondence between the placement of the dots along respective directions in the plots in this figure and the corresponding plots in Fig. 6. The colour palette depicts first passage times in hours. The 0 degree direction represents east with respect to the actual landscape. Blue crosses show the location of repulsive features that are considered in computation of first-passage times. (**a**) GH1Br, (**b**) GH3Br, (**c**) GH4Br, (**d**) GH2NBr, (**e**) GH5NBr, (**f**) WH1Br.

Some general features were also evident from the first passage time analysis (Figs. 6, 7). $\alpha = 8$ gave a good sense of search times compared to other alternatives for all hornbills irrespective of their breeding status and time of the year (see the Section “Calculating first passage times” and Table 3 in Methods). The angles shown in Fig. 7 increase anti-clockwise from 0 degrees which corresponds to the east direction.

**Figure 8:**
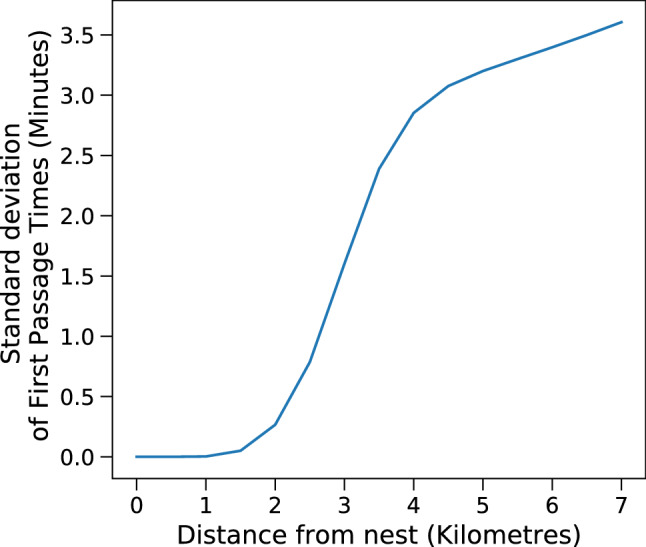
Standard deviation of first passage times for WH1Br at different distances from its nest. At a given distance, the standard deviation is obtained over all the movement directions. The region of steepest increase represents areas around human settlements. The standard deviation saturates after a certain distance since the bird has crossed the human settlements along the directions where they exist.

For a given bird, first passage times were mostly identical in different directions at short distances from the roosting/nesting sites. Search times were consistently higher in directions where human modified landscapes existed, more so if these were located at short distances from the bird’s roost/nest. This is especially evident for GH1Br and GH5NBr since their home range boundaries lie close to human settlements. Wreathed hornbills are known to move over large distances to track fruit resources at large scales^15^. WH1Br exhibited much more varied movement patterns, as is expected of a Wreathed hornbill. It also ventured into the forested areas beyond the human settlements. The first passage times for WH1Br increase more in directions of settlements initially. WH1Br reached areas beyond the human settlements after some distance, and this was reflected in the standard deviation of first passage times. The standard deviation in first passage times increased with distance up to a point beyond which it saturated (Fig. 8). This is evident in Fig. 8 and the movie file SI Fig 4 linked to the figure in Supplementary Information. In contrast, if human-modified landscapes were sufficiently far from a given bird, their search times increased almost isotropically from the starting location, which is the case for GH3Br. Overall, an anisotropic rise in first passage times was only noticed near forest edges that lie closer to human settlements.

## Discussion and conclusions

Our framework is very general and would also hold for other animals that live in fairly resource rich environments. Modelling movement in landscapes with sparse resources is also possible given a fair knowledge of resource distribution. Here, we model the probability distribution of space use for hornbills and drift and diffusion coefficients were calculated from recorded data.

The breeding hornbills are bound to access a much smaller area around their nesting sites, and thus make numerous trips between their nests and fruiting trees. A higher value of *D* is a reflection of the repeated movement of these birds over recently traversed paths by random switches in the direction^11^.

For a given species, the ratio of coefficients (R) has a particular range that further shows a clear partition between breeding and non-breeding birds. We suggest that in the absence of visual observations, the ratio R obtained from telemetric data, may be used as an additional quantifier for the breeding status of individuals in a given species.

The analysis suggests that seasonal variability in foraging resources/fruit availability has a low influence on movement patterns of non-breeding hornbills. The ratios for the 2 non-breeding birds were very similar even though their data pertains to different times of the year. It must be noted that non-breeding hornbills are not constrained in terms of just staying around one nest, and can therefore access multiple resource-rich areas. In fact, we could identify at least three locations for both non-breeding hornbills from data where they stayed for two consecutive nights or more. So, once they reach these locations, they can obtain optimal nutrition by moving minimally. Although they range farther than breeding birds, the distances covered by them per day are relatively smaller^14^. Also, since non-breeding individuals do not need to return to a nest site frequently, they can search for resources spread over larger areas. We do not disregard the possible influence of resource availability on the choice of roosts around which these birds forage. However, once this choice is made, the directedness of motion or random switching of directions has a weak dependence on the fruit availability around the roosts.

The recorded data for GH4Br spans only 20 days, in contrast to other individuals that were tracked for more than 50 days. The estimated values of its coefficients are less reliable since they reflect only a small part of the breeding season. This could explain the slight departure of its ratio R from that for GH1Br and GH3Br.

GH1Br is the only individual among the Great hornbills that crossed the Pakke river and accessed some parts of the human modified landscape (Fig. 2). This could be the case because its nest is very close to the river and Great hornbills may be facultatively territorial during the breeding season, or at least defend the nest area and its immediate vicinity. It is possible that this bird avoided certain patches within the forested side because of the nests of other Great hornbills there. More information about resource distribution at finer scales could help account for this observation. On the other hand Wreathed hornbills have different dietary needs and foraging strategies and are non-territorial^15,16^. Moreover, even though it is a smaller body-sized species than the Great hornbill, it ranges over larger areas. WH1Br shows much less constrained movement patterns for a breeding hornbill. It is known to have multiple roosting sites, and accessed forested areas lying to the east of Pakke river and the adjoining human settlements outside the Tiger Reserve.

Our model captures the probability distribution of spatial use by the hornbills over long times. In doing so, it uses minimal information such as the locations of roosts/nests.The steady state probability distributions give information about space use that is sub-optimally captured by analysis of movement trajectories and home range boundaries. In our work, we only use information about nests/roosts to find these probability distributions of long term space use. In case of just a single nest, this yields homogeneous probabilities of finding a bird at fixed radius from the nest. The predicted distributions are more heterogeneous for the non-breeding birds as a consequence of multiple roost sites that we used in the analysis. The heterogeneities in space use at finer scales –irrespective of the breeding status—could be identified using other kinds of information such as distribution of fruiting trees. This would be ensured by the fact that our model predicts multi-modal distributions in case there are multiple centres of attraction in a landscape. We leave the inclusion of resource distribution for future work.

Our study shows that first passage times increase with proximity to human settlements, especially if these are located at short distances from a bird’s nest/roost. This is expected since an individual cannot search optimally in these areas, more so when it comes closer to them. Human-modified landscapes may constrain the great hornbills in foraging efficiently within their small home range when they breed, and they would potentially have to spend more time in searching for food. Hornbills have a preference for specific nest tree species with certain characteristics and such trees are limiting^17^, however they do use nest trees and breed successfully outside protected areas. However, the extent and degree of human modification around these sites determine whether such nest trees are occupied and successful^18^. While nesting may happen even at degraded sites, foraging movements may be lengthened or constrained due to poor resource availability and human disturbance in the modified landscapes outside^19^. A detailed knowledge of their nesting sites would therefore be very useful in informing land development decisions, especially outside protected areas.

## Methods

### Information on hornbill data

#### Study area

The study was carried out in Pakke Tiger Reserve (PTR; area: $861.9\,\hbox {km}^2$; elevation: 150–1800 m ASL; range: $92^{\circ }36'$ –$93^{\circ }09'\hbox {E}$ and $26^{\circ }54'$–$27^{\circ }16'\hbox {N}$), a protected area, in Arunachal Pradesh state which is part of the Eastern Himalaya Biodiversity Hotspot. The intensive study site was in the south-eastern corner of the reserve. The vegetation is classified as Assam Valley tropical semi-evergreen forest^20^. More than 78% of trees are biotically-dispersed^21^. Human settlements lie distributed along the Pakke river which flows along the south-eastern boundary of the reserve.

#### Hornbill species

The body size of the Great Hornbill is 2.2–4 kg, while that of the Wreathed hornbill is 1.4–3.7 kg^22^. The hornbill breeding season in this area is from March to mid-August. IUCN has classified the Great and Wreathed Hornbills as ‘Vulnerable’ (IUCN 2018)^23^.

### Field methods

#### Hornbill movement

We (RN and AD) tagged five adult, male Great Hornbills and one adult, male Wreathed Hornbill between October 2014 and May 2016. We trapped the hornbills using canopy-mounted mist nets at fruiting fig trees. The captured birds were measured, weighed and tagged. Only adult male birds were fitted with battery-operated e-obs GPS loggers (Model ‘Bird 1A’). Tags were produced by e-obs GmbH (https://www.e-obs.de, Germany). The weight of the tag was 55 gm which is less than 2% of the weight of Wreathed and Great Hornbills. The tag was fitted like a backpack using Teflon strings (0.55” wide). We did not tag female and juvenile birds as they could affect female entry/exit at nests or the growth of juvenile birds. The data loggers took GPS fixes at 15-minute intervals between 3 am and 7 pm (one hour before and after sunset in this area). The stored data was remotely downloaded using a base station.

### Ethics statement

We (RN and AD) obtained research and animal capture permits from the Arunachal Pradesh Forest Department, National Tiger Conservation Authority and the Ministry of Environment and Forests, New Delhi and conducted the research under the supervision of Pakke Tiger Reserve management. Ethics clearance was obtained from the Ethics Committee of the Nature Conservation Foundation. We followed established methods of capture/tagging of hornbills and were advised by senior wildlife veterinarian Dr.Parag Deka, Aaranyak to minimize risk to individual birds.

### Fitting step-lengths and angles to distributions

We chose a heuristic to filter the recorded data available. We computed step-lengths corresponding to every two consecutive coordinates. If the sum of tag GPS-errors for a pair of consecutive coordinates was greater than the corresponding step-length, we did not use them in our analysis. An alternative was to exclude all step lengths that lie below the average GPS-error of the tag. However, this is excessive since many of these small step lengths correspond to locations with lower GPS-errors than average and therefore should not be excluded.

**Figure 9:**
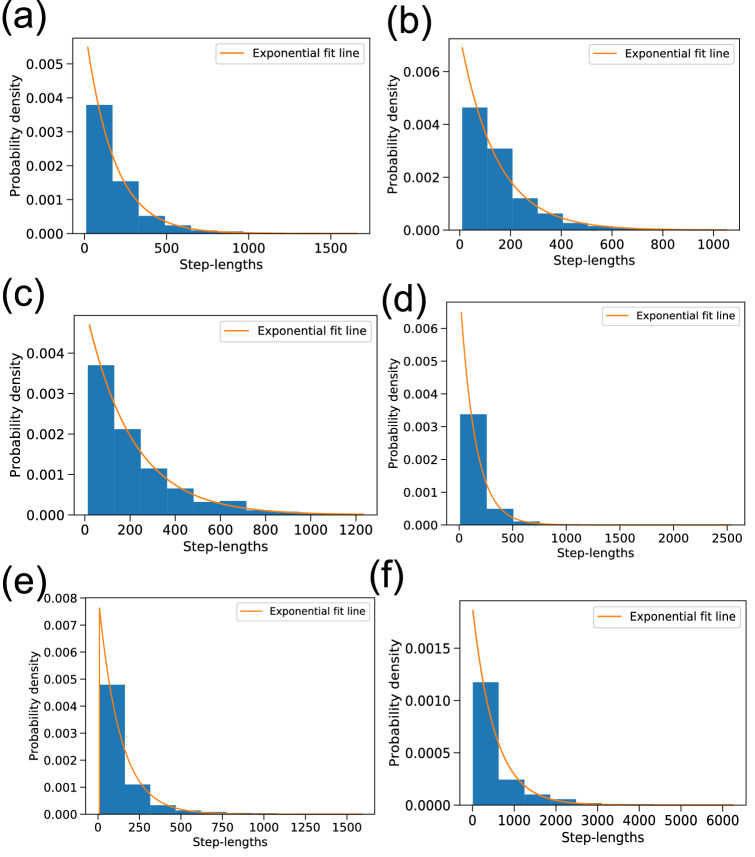
Best fit lines corresponding to exponential distribution of step lengths for different hornbills. (*p*-values from KS test for (**a**) GH1Br (${p} = 0.0010$), (**b**) GH3Br ($p = 0.0005$), (**c**) GH4Br ($p = 0.0755$), (**d**) GH2NBr ($p = 0.0028$), (**e**) GH5NBr ($p \le =10^{-10}$) and (**f**) WH1Br (${p}\le 10^{-10}$).

We checked the step-length distributions for all hornbills against exponential distributions first. We used the Kolmogorov-Smirnov^24,25^ (KS from here onwards) test statistic to assess the goodness of fit. A high p-value indicated that the null hypothesis—the two distributions being compared are identical—cannot be rejected. The test displayed in Fig. 9 did not show a good fit for GH5NBr and WH1Br. We tried other distributions such as Weibull and Beta distribution as well but we got poorer or similar fits in most cases.

**Figure 10:**
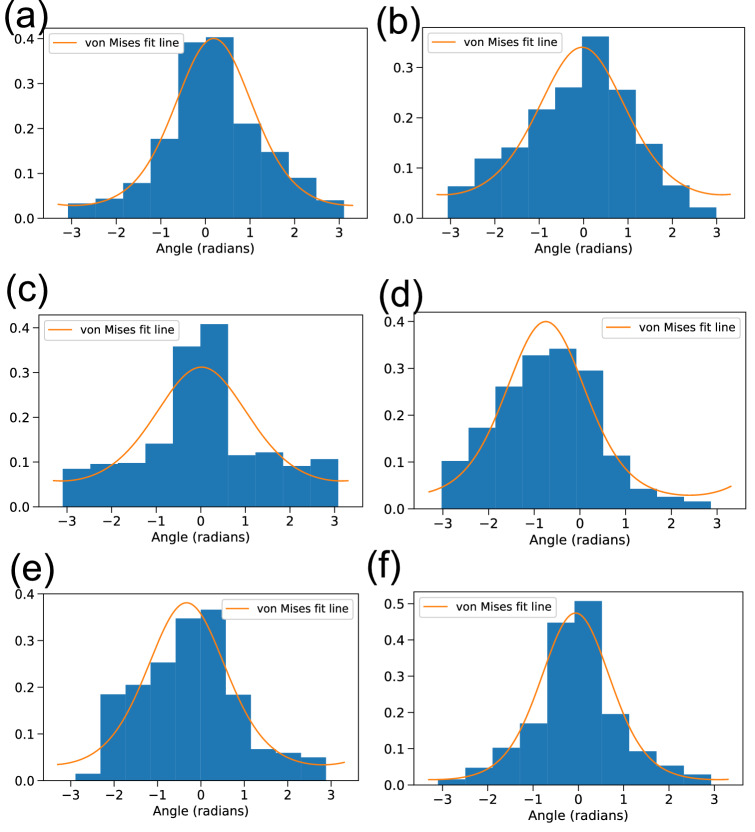
Best fit lines from von Mises distribution against histograms of angles for different hornbills. (p-values from KS test for (**a**) GH1Br (${p} = 1.20 \times 10^{-7}$), (**b**) GH3Br (${p} = 0.0035$), (**c**) GH4Br (${p} = 0.002$), (**d**) GH2NBr (${p} = 0.016$), (**e**) GH5NBr (${p} = 6.07\times 10^{-5}$), (**f**) WH1Br ${p} \le 10^{-10}$).

We computed the angle corresponding to every step with reference to a point of attraction, which was the nest for breeding birds or the most frequented roost otherwise. We expected the distribution of such angles to follow a von Mises distribution, which is also alluded to in the literature^12^. The von Mises distribution is a circular distribution which is similar to the normal distribution in linear statistics^26^. This is useful for angles which could be used in periodic functions to yield the same values over different domains. We tested the fit to this distribution using KS test again (Fig. 10). The $\kappa$ parameter in this distribution quantifies the concentration of the distribution around some mean direction. This is particularly useful for situations where one is interested in bias towards a particular feature. We computed $\kappa$ with reference to two most frequented roosts for each of the non-breeding hornbills. The roosts used by GH2NBr were at least 15 kilometres apart but yielded very similar values of the parameter—1.31 and 1.38. This parameter encodes the general non-uniformity of movement directions, which does not vary much across preferred sources of attraction.

The process of filtering the data improved the fit to claimed distributions for some of the birds. Our heuristic did not rely on placing a cut-off in terms of removing step-lengths below a certain number (Eg: Average GPS-error), so a good number of small step-lengths were also retained. The two-dimensional probability distributions that we generated from data had large grids (two-dimensional bins) which means that the filtering process had a very small effect on the mean-squared displacement.

### Probability distribution of space use of territories of other hornbills

**Figure 11:**
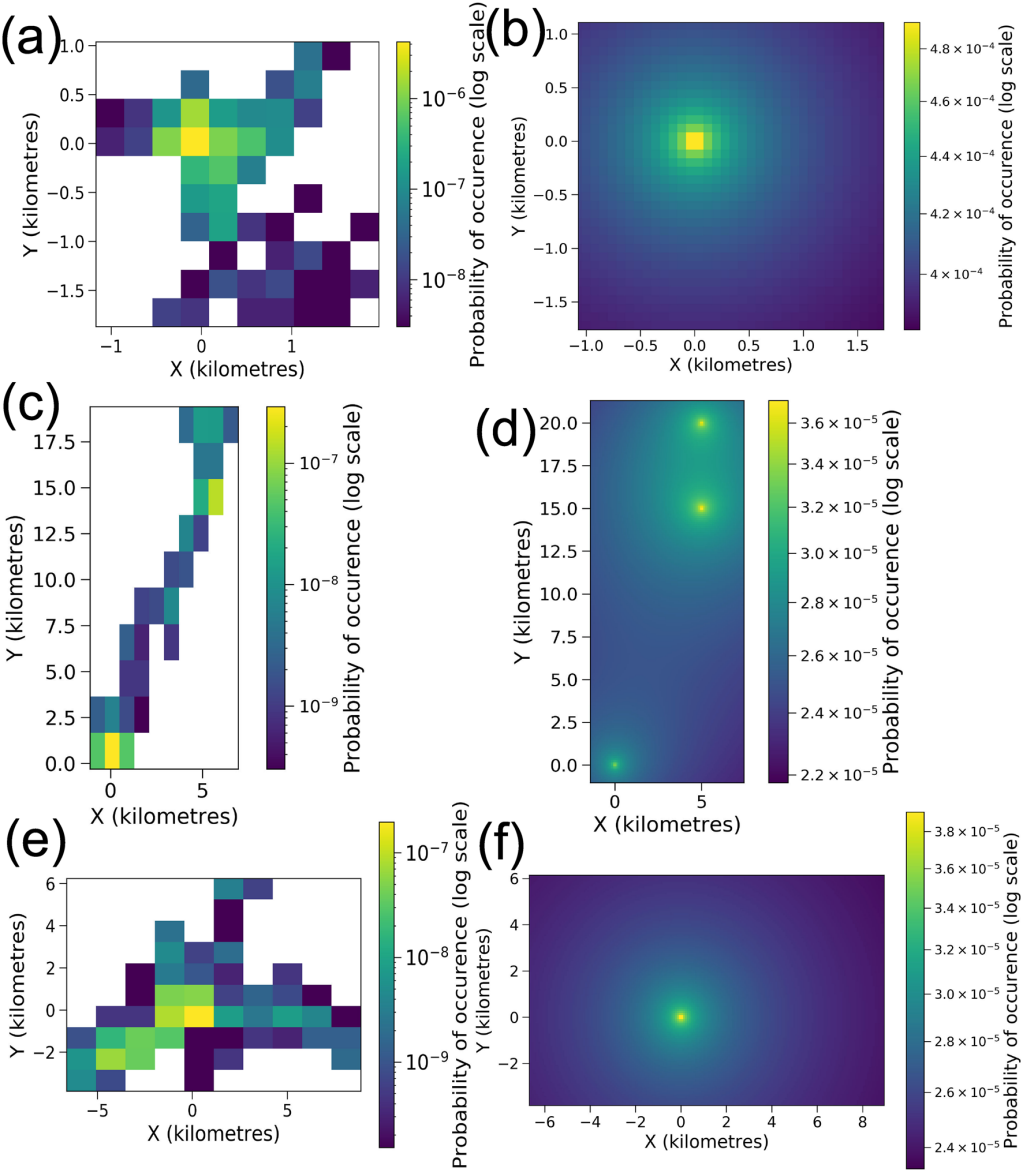
Visualization of the probability of space use over the territories from empirical data (plots on the left) against the steady-state probability distributions from Fokker–Planck equation (plots on the right). X and Y distances along the respective axes are in kilometres. The empirical plots were generated from 2-d histograms of location data. The location data was first projected to UTM coordinates. The origin was fixed at the nest for breeding birds and at one of the roosting sites for non-breeding hornbills. The smaller grid size is necessary to get greater accuracy in the numerically generated solutions. The numerical solutions also demand axes that are symmetric around the origin. Therefore, the histogram based probability distributions from data have very different grid sizes for their visualizations. The plots based on numerical solutions have been cropped to match the extent of axes of those from empirical data. (**a**, **b**) GH1Br, (**c**, **d**) GH2NBr, (**e**, **f**) WH1Br.

We calculated the probability distributions of space use for the hornbills from observed data and compared these against the steady state distributions obtained from the Fokker–Planck equation, in Fig. 11.

### Calculating first passage times

We converted our First Passage Time equations into polar form since boundary conditions were easier to define in this coordinate system. We used the forward difference formula for spatial derivatives in the discretized version of Eq. (12). These discrete forms could be read off from the terms involving $\Delta r$, $\Delta r^2$ and $\Delta \theta ^2$ in Eq. (13). Another option that we tried was using a forward difference formula for first order spatial derivatives but a central difference formula for derivatives of second order. We checked the efficiency of both choices by comparing values of first passage times at 0 and 360 degrees at the same radius. We noticed that they were almost the same when using just the forward difference formulae. The other possibility showed a larger deviation and was thus ruled out.

The first passage time equation in (12) was converted into polar form and discretized to give:

13 $$\begin{aligned}&\frac{2\alpha }{r}\gamma \times \Big ( \frac{T_{p+1}^q - T_{p}^q}{\Delta r} \Big ) \nonumber \\&\quad -\,\gamma \sum _{j} \left\{ r e^{-(r^2 + r_{j}^2 - 2r \times r_{j} \times cos(\theta - \theta _{j}))} \right\} \times \Big ( \frac{T_{p+1}^q - T_{p}^q}{\Delta r} \Big )\nonumber \\&\quad +\, D \Big [ \frac{T_{p+1}^q - 2T_{p}^q + T_{p-1}^q}{(\Delta r)^2} + \frac{1}{r} \Big ( \frac{T_{p+1}^q - T_{p}^q}{\Delta r} \Big ) \nonumber \\&\quad +\, \frac{1}{r^2} \Big ( \frac{T_{p}^{q+1} - 2T_{p}^q + T_{p}^{q-1}}{(\Delta \theta )^2} \Big ) \Big ] + 1= 0 \end{aligned}$$

where summation label *j* runs over all the repelling features. We only considered attraction to a single location here which was set as the origin, since other roosting sites would not play a role in influencing the hornbills when they search for a target within a day. The indices *p* and *q* correspond to the $p^{th}$ radius and $q^{th}$ angle respectively at any particular point $(r,\theta )$ of the grid where points are represented in polar coordinates. Our equation can be put in a matrix form as:

14 $$\begin{aligned} AT_{p+1} = BT_{p} - r_{3}r^{2}T_{p-1} + r^{2}r_5 \end{aligned}$$

A and B are $N \times N$ square matrices where N is the number of angles and radii that we want to create grids. $T_{p}$ is a vector of size N.

$$\begin{aligned} A= \left[ {\begin{array}{ccccc} a_{11} &{} 0 &{} ... &{} ... &{} 0 \\ 0 &{} ... &{} ... &{} ... &{} ... \\ ... &{} ... &{} ... &{} ... &{} ... \\ ... &{} ... &{} ... &{} ... &{} ... \\ 0 &{} ... &{} ... &{} 0 &{} a_{11} \\ \end{array} } \right] \end{aligned}$$

Matrix A has the same value across all diagonal elements. Off diagonal entries are 0. Also, $a_{11} = r_2r - r_6 r^3 \sum _{j} exp(-( r^2 + r_{j}^2 - 2r \times r_{j} \times cos(\theta - \theta _{j})) + r_3 r^2 + r_4 r$.

$$\begin{aligned} B= \left[ {\begin{array}{ccccc} b_{11} &{} -k_1 &{} 0 &{} ... &{} 0 \\ -k_1 &{} ... &{} -k_1 &{} ... &{} ... \\ ... &{} ... &{} ... &{} ... &{} ... \\ ... &{} ... &{} ... &{} ... &{} ... \\ 0 &{} ... &{} 0 &{} -k_1 &{} b_{11} \\ \end{array} } \right] \end{aligned}$$

Matrix B also has the same entry for all diagonal elements, i.e., $b_{11} = r_2r - r_6 r^3 \sum _{j} exp(-( r^2 + r_{j}^2 - 2r \times r_{j} \times cos(\theta - \theta _{j})) + 2r_3 r^2 + r_4 r + 2k_1$. All rows except the first and last have $-k_1$ as an entry just before and after the diagonal element.

$$\begin{aligned} T_{p} = \left[ {\begin{array}{c} T_{p}^0 \\ T_{p}^1 \\ ... \\ ... \\ ... \\ \\ \\ T_{p}^{N-1}\\ \end{array} } \right] \end{aligned}$$

Here, $$k_1=D(\Delta r)^2$$;$$k_2=2\alpha \times \gamma (\Delta r)(\Delta \theta )^2$$; $$k_3=D(\Delta \theta )^2$$ $$k_4 = D(\Delta r) (\Delta \theta )^2$$; $$k_5 = (\Delta r)^2(\Delta \theta )^2$$; $$k_6 = \gamma (\Delta r) (\Delta \theta )^2$$.

$\Delta r$ and $\Delta \theta$ are radial and angular grid spacings which depend on the number of grid points.

**Table 3:** Hornbill first passage times at fringes of home range for $\alpha =8$.

Angles (degrees)	First passage times (in hours)					
	GH1Br	GH4Br	WH1Br	GH3Br	GH2NBr	GH5NBr
0	0.257	1.042	0.897	0.065	7.614	3.900
25.714	0.207	0.944	0.867	0.064	7.614	3.816
51.429	0.204	0.935	0.736	0.064	7.614	3.816
77.143	0.203	0.935	0.734	0.064	7.614	3.816
102.857	0.203	0.935	0.734	0.064	7.614	3.816
128.571	0.203	0.935	0.734	0.064	7.614	3.816
154.286	0.203	0.935	0.734	0.064	7.614	3.816
180	0.203	0.935	0.734	0.064	7.614	3.816
205.714	0.203	0.935	0.735	0.064	7.614	3.816
231.429	0.203	0.935	0.738	0.064	7.614	3.811
257.143	0.208	0.938	0.747	0.064	7.614	4.910
282.857	0.229	1.091	0.774	0.064	7.588	5.702
308.571	0.289	1.715	0.813	0.064	17.106	4.388
334.286	0.307	0.998	0.780	0.064	7.817	3.833
360	0.257	1.042	0.901	0.065	7.614	3.900

First passage times calculated with $\alpha =8$ are shown in Table 3 for hornbills. These may be compared with those obtained for $\alpha =4$ and 2 displayed in Supplementary Tables 1, 2. We find that the calculated values are closest to recorded data for $\alpha =8$. These first passage times were computed at the farthest extent of each hornbill’s home range in different directions. GH3Br had a very small home range of 1–2 sq.km. The frequency of our data is 15 minutes but it is evident from the data that GH3Br took less than that duration to reach the fringes of its home range. $\alpha =8$ has times that are sufficiently below that value whereas $\alpha =4$ and $\alpha =2$ predict higher times (see Supplementary Tables 1, 2). The first passage times become highly unrealistic for lower values of $\alpha$ which is evident from the $\alpha =2$ case. In general, predicted values for $\alpha <8$ are higher than what is roughly expected. Similarly, $\alpha >8$ yields lower first passage times which are not consistent with the patterns observed for all hornbills.

## Supplementary Information

Supplementary Video 1

Supplementary Video 2

Supplementary Information 3

## Data Availability

The hornbill data that support the findings of this study are available at^14,27^.

## Code availability

Requests related to the numerical modelling code associated with this paper should be sent to the authors.
