# Supplementary material for: The flight of the hornbill: drift and diffusion in arboreal avian movement
Source: Sci Rep. 2021 Mar 10;11:5591. doi: 10.1038/s41598-021-84074-3 (PMC7946904; doi:10.1038/s41598-021-84074-3)
Supplement: Supplementary file 3 — Supplementary Information 3 [file 41598_2021_84074_MOESM3_ESM.pdf]

Supplementary information: The flight of the hornbill: drift and diffusion in arboreal avian movement

Ankit Vikrant[1], Janaki Balakrishnan[1,*] Rohit Naniwadekar[2], and Aparajita Datta[2]
[1]*School of Natural Sciences and Engineering, National Institute of Advanced Studies (N.I.A.S.), Indian Institute of Science Campus, Bangalore - 560012, India and*
[2]*Nature Conservation Foundation, 1311, Amritha, Vijayanagar 1st Stage, Mysore - 570017, India.*

Details of numerical computations of first passage times

In eqns.(7,8), κ_τ is determined using the 'est.kappa' function from 'CircStats' library in R. It simply finds maximum likelihood estimate of kappa from turning angle data. ρ_τ was similarly evaluated by taking inverse of the rate parameter of exponentially distributed step length data. 'eexp' function from the package 'EnvStats' was used for maximum likelihood estimation of the rate parameter.

Equation (11) is a linear system of equations that we solve at each radius step using *linalg* package in numpy. All first passage times are set to 0 at $r = 0$. It must be noted that the solutions are sensitive to the grid spacings Δr and $\Delta\theta$. The values of Δr and $\Delta\theta$ should not be too small else the solutions misbehave. This is because at the solution at every radial step are approximations. These errors accumulate as we progress with more number of steps. The algorithm used performs well even for upto 30 angular and radial points in many cases. To keep errors to a minimum, it is recommended that the grid should not have more than 15 angles and 15 radii. A good way to check the validity of solutions is to compare the first passage time values obtained at 0 and 360 degrees. Our solutions do well if the values at 0 and 360 degrees are roughly the same. Calculation of the first passage time T (from equation (9)) requires knowledge of the value of α in the expression for the potential (eqn.(3)). This is determined in the following manner. Equation (9) is first solved for T using some arbitrary α value at the farthest home range extent for each bird in different directions. The value so obtained is compared with actual recorded telemetry data. If the calculated values differ significantly from or are larger than the recorded values, then we consider the corresponding α value as inappropriate.

Supplementary Tables:

Angles (degrees)	First Passage Times(in hours)					
	GH1Br	GH4Br	WH1Br	GH3Br	GH2NBr	GH5NBr
0	0.749	2.323	3.369	0.131	15.026	7.862
25.714	0.471	1.846	2.813	0.126	15.026	7.528
51.429	0.413	1.817	1.272	0.126	15.026	7.533
77.143	0.402	1.817	1.420	0.126	15.026	7.533
102.857	0.401	1.817	1.416	0.126	15.026	7.533
128.571	0.400	1.817	1.416	0.126	15.026	7.533
154.286	0.400	1.817	1.416	0.126	15.026	7.533
180	0.400	1.817	1.417	0.126	15.026	7.533
205.714	0.401	1.817	1.419	0.126	15.026	7.533
231.429	0.403	1.817	1.431	0.126	15.026	7.484
257.143	0.418	1.826	1.458	0.126	15.026	6.853
282.857	0.517	2.683	1.581	0.126	15.049	525.296
308.571	1.864	1.226	2.227	0.126	8.064	4.746
334.286	-198.097	2.058	1.420	0.126	16.045	7.751
360	0.734	2.320	3.50	0.131	15.025	7.862

SUPPLEMENTARY TABLE - 1: Hornbill first passage times at fringes of home range for $\alpha = 4$.

*Corresponding author email: janaki05@gmail.com

Angles (degrees)	First Passage Times(in hours)					
	GH1Br	GH4Br	WH1Br	GH3Br	GH2NBr	GH5NBr
0	-9.605	6.798	-105.682	0.263	29.274	15.966
25.714	1.001	3.527	37.662	0.242	29.274	14.64
51.429	0.821	3.435	-0.689	0.243	29.274	14.685
77.143	0.782	3.437	2.760	0.243	29.274	14.683
102.857	0.777	3.437	2.642	0.243	29.274	14.683
128.571	0.777	3.437	2.643	0.243	29.274	14.683
154.286	0.776	3.437	2.642	0.243	29.274	14.683
180	0.776	3.437	2.647	0.243	29.274	14.683
205.714	0.777	3.437	3.476	0.243	29.274	14.681
231.429	0.785	3.436	-1.992	0.243	29.274	15.808
257.143	0.841	3.452	-7.322	0.243	29.274	-162.963
282.857	-1.718	227.505	184.086	0.243	29.317	-4.244
308.571	1.711	1.241	-108.459	0.243	19.898	1.243
334.286	-9.111	4.546	-327.921	0.242	33.896	15.879
360	-9.605	6.761	-105.682	0.263	29.27	15.953

SUPPLEMENTARY TABLE - 2: Hornbill first passage times at fringes of home range for $\alpha = 2$.
